# Large Variations in Declared Serving Sizes of Packaged Foods in Australia: A Need for Serving Size Standardisation?

**DOI:** 10.3390/nu10020139

**Published:** 2018-01-28

**Authors:** Suzie Yang, Luke Gemming, Anna Rangan

**Affiliations:** Nutrition and Dietetics, School of Life and Environmental Sciences, Charles Perkins Centre, The University of Sydney, NSW 2006, Australia; syan1164@uni.sydney.edu.au (S.Y.); luke.gemming@sydney.edu.au (L.G.)

**Keywords:** dietary guidelines, food labelling, food legislation, portion size, serving size

## Abstract

Declared serving sizes on food packaging are unregulated in Australia, and variations in serving size within similar products reduces the usability of this information. This study aimed to (i) assess the variations in declared serving sizes of packaged foods from the Five Food Groups, and (ii) compare declared serving sizes to the Australian Dietary Guidelines standard serves and typical portion sizes consumed by Australian adults. Product information, including serving size, was collected for 4046 products from four major Australian retailers. Within product categories from the Five Food Groups, coefficients of variation ranged from 0% to 59% for declared serving size and 9% to 64% for energy per serving. Overall, 24% of all products displayed serving sizes similar (within ±10%) to the standard serves, and 23–28% were similar to typical portion sizes consumed by adults, for females and males, respectively. In conclusion, there is substantial variation in the declared serving sizes of packaged foods from the Five Food Groups, and serving sizes are not aligned with either the Dietary Guidelines or typical portion sizes consumed. Future research into effective means of standardising serving sizes is warranted.

## 1. Introduction

Nutrition information labelling on packaged food items is a valuable source of information that can assist consumers to engage in more informed food choices and consumption behaviours. In Australia, most packaged foods must adhere to nutrition labelling standards set out in the Australia New Zealand Food Standards Code [1], which are enforced by law. Nutrition information panels (NIPs) on food packaging are required to display the serving size and number of servings per package of the food item, as well as the energy (in kilojoules/kJ) and key nutrients, both ‘per serving’ and ‘per 100 g’ (or 100 mL for liquids) of the food [2]. Manufacturers have access to online tools to calculate the nutrition information of their products based on the types and amounts of ingredients included. The intended purpose of per serving nutrition information is to help consumers estimate the amounts of energy and nutrients that are being consumed, while nutrition information per 100 g/100 mL can be used to compare similar products [2].

A prerequisite for the efficacy of nutrition labelling is that consumers understand how to interpret and apply this information appropriately. However, evidence shows that the use of serving size and per serving nutrient information is poorly understood by consumers [3,4,5,6]. In Australia, this is likely due, in part, to a lack of regulation regarding declared serving sizes, as the quantity of food that constitutes a serving is determined by the food manufacturer [7]. While it is suggested that declared serving sizes “should reflect a realistic portion of the food that a person might normally consume on one eating occasion” [7] (i.e., a realistic portion size), there are no official guidelines specifying what a realistic portion of different foods may be. As such, declared serving sizes and energy per serving can vary considerably between similar products [2]. This makes it difficult for consumers to effectively monitor and/or regulate their intake, and has led to suggestions to standardise serving sizes within food categories [8,9], as occurs in the USA and Canada [10,11].

Another potential source of consumer confusion is the discrepancy between manufacturer-declared serving sizes and the standard serves for different foods [4,12,13], as defined in the Australian Dietary Guidelines (ADG) [14,15,16]. The standard serves form part of a guide to help individuals choose appropriate daily amounts of different types of foods for optimal health and wellbeing. Such discrepancies may therefore confuse consumers who are trying to follow official dietary recommendations [12].

Previous Australian studies have found large variations in declared serving sizes and energy per serving within discretionary and snack food categories [14,15,17]. However, there are currently limited data on serving size variation within product categories from the Five Food Groups. This study aimed to assess the variation in serving sizes and energy per serving across packaged food categories from the Five Food Groups. A second aim was to compare the manufacturer-declared serving sizes with the ADG standard serves, and with sex-specific typical portion sizes consumed by Australian adults, as estimated from the 2011–12 National Nutrition and Physical Activity Survey (NNPAS) [18].

## 2. Materials and Methods 

Data were collected between March and September 2017 for packaged food items available in four major supermarkets (Woolworths, Coles, Aldi, and IGA) in Sydney, Australia. Collectively, these retailers represent 92% of the grocery market share in Australia [19]. Images were captured of the front, back, NIP, ingredients list, and barcode of each product, using Android smartphones (Motorola Moto G4 Play). Different package sizes of the same product were included as separate items, as the declared serving sizes can differ. Products without an NIP were not included. Product information, including declared serving size and energy (kJ) per 100 g/100 mL, were recorded in an online spreadsheet.

Data cleaning included removal of duplicate items and screening for outliers, which were verified using the associated product images. Items missing serving size information (*n* = 7) or with apparent labelling error (*n* = 11) were excluded from analyses. For items available in multi-unit packages of different sizes (e.g., 12-pack vs. 24-pack sliced cheese), only one was included in analyses, as declared serving sizes are the same.

Products were categorised largely according to the ADG classification of foods within the Five Food Groups [16], with further sub-groupings based on expected differences in serving size, energy density and/or other nutrient composition (e.g., full- vs. reduced-fat milk). A summary of product types included in each category is provided in Appendix A Table A1. For the purpose of this study, legumes were categorised under the vegetables food group [16]. Energy per serving was calculated for all products from declared serving size and energy per 100 g/100 mL. To assess the variations in serving size, descriptive statistics (mean, standard deviation (SD), median, interquartile range (IQR), and range) for declared serving size and energy per serving were generated for each product category. Coefficients of variation (CV) were calculated using SD divided by the mean, to obtain a standard measure of variation across categories.

Declared serving sizes were compared to the ADG standard serves by calculating the percentage difference between the median declared serving size of a category and the corresponding standard serve. A difference of >25% was considered to be substantial. As per the method used by Zheng et al. [18], percentage differences were calculated as follows:

(1) $$\frac{\left(median\;declared\;serving\;size\;\;standard\;serve\right)}{standard\;serve}\;\times \;100$$

The proportion of products within each category with a declared serving size >10% below, within ±10%, or >10% above the corresponding ADG standard serve were assessed, with ±10% being taken as an ‘ideal’ range of variation about the standard serve. In the same manner, declared serving sizes were compared to sex-specific typical (median) portion sizes of foods consumed by Australian adults [18]. Portion sizes for liquids (i.e., fruit juice, milks), given in grams, were converted to millilitres using density measures provided in the AUSNUT 2011–13 food measures file [20]. A list of the categories [18] used for portion size comparison are displayed in Appendix A Table A2.

Statistical analyses were performed using IBM SPSS Statistics Version 24 (IBM Corp., Armonk, NY, USA, 2016). Ethics approval was not required for this study.

## 3. Results

A total of 4046 products, in 39 categories, across the Five Food Groups, were included (Table 1). The CV for declared serving size ranged from 0% to 59% across product categories. Categories with the least variation (CV < 10%) were plain dairy and non-dairy milks, while those with the most variation (CV > 50%) were tofu and cottage-/ricotta-style cheeses. Within several categories, 10-fold (or greater) differences were observed between the smallest and largest serving sizes (e.g., crispbreads, canned vegetables, nuts and seeds, and some cheeses). Energy per serving was more variable than declared serving size, with CV ranging from 9% to 64%. The largest variations (CV > 50%) were observed for frozen vegetables, canned vegetables, other seafood, tofu, and cottage-/ricotta-style cheeses.

Table 2 shows the comparison between median declared serving sizes and the ADG standard serves, as well as typical (median) portion sizes consumed by Australian adults. Thirty-seven of the 39 categories analysed had corresponding ADG standard serves (dry porridge oats and meat substitutes excepted). The median declared serving sizes for children’s cereals, frozen fruit, nuts and seeds, and all milks were equivalent to the ADG standard serves. Conversely, 21 out of 37 categories had median serving sizes that were substantially different (>25% difference) to the standard serve. Differences between median declared serving sizes and typical portion sizes were examined for 28 comparable categories. Median serving sizes for 15 out of 28 categories were substantially different (>25% difference) to the typical portion size for one or both sexes. Seven out of 28 categories showed substantial differences for both sexes; these categories were all within the meat and dairy food groups.

**Figure 1:**
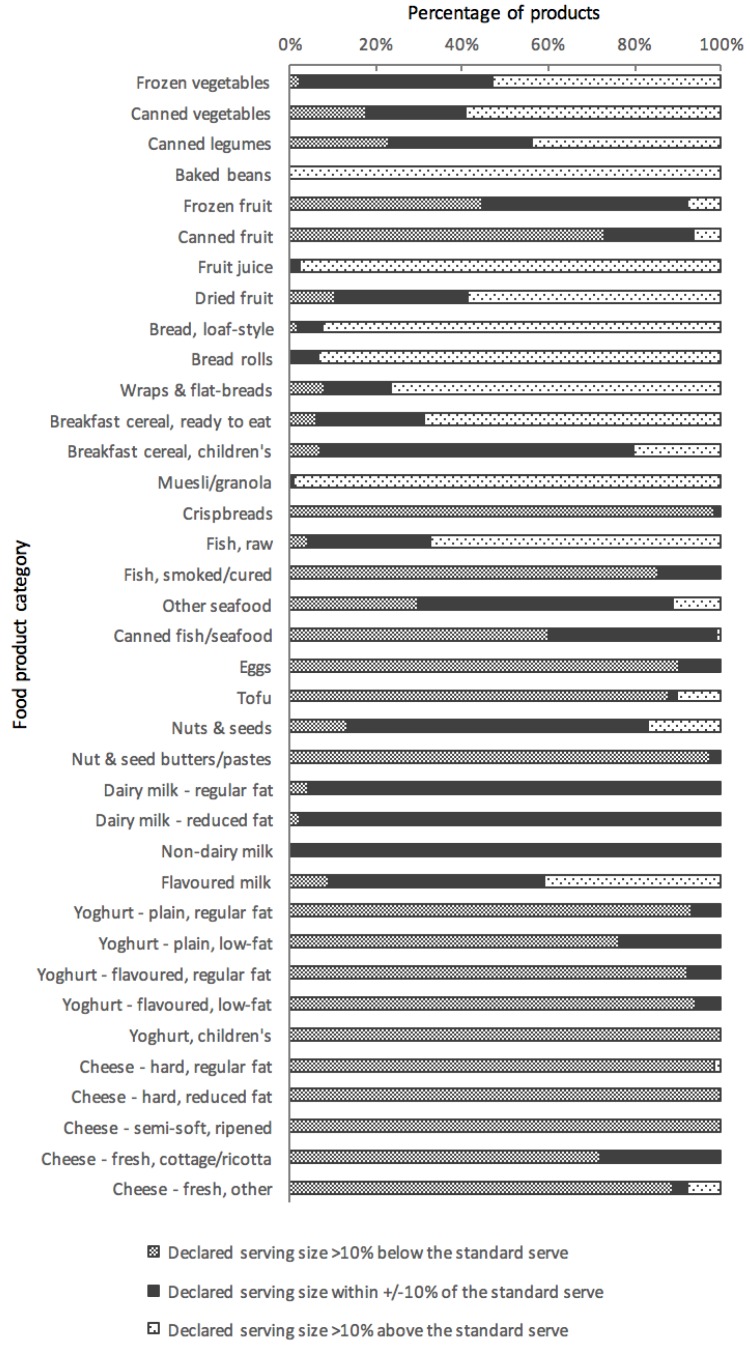
Distribution of products within each food category from the Five Food Groups according to the similarity of their declared serving size to the corresponding Australian Dietary Guidelines (ADG) standard serve. Note: the category ‘Breakfast cereal, ready to eat’ excludes children’s cereals and muesli/granola.

Figure 1 displays the distribution of products within each product category according to the similarity of their declared serving size to the corresponding ADG standard serve. Seven out of 37 categories contained a majority (≥50%) of products with declared serving size similar to (i.e., within ±10% of) the ADG standard serve. Serving sizes for plain dairy and non-dairy milks were the most consistent with the standard serves. In contrast, 17 out of 37 categories contained very few products (≤10%) with a serving size similar to the standard serve. Overall, 24% (948/3898) of all products displayed a declared serving size similar to the corresponding ADG standard serve. In general, declared serving sizes of products within vegetable and grain (cereal) food categories were substantially greater than the ADG standard serves, while those within meat (canned fish, eggs, tofu, nut and seed pastes) and dairy categories (yoghurts and cheeses) were substantially less than the standard serves, and products within fruit categories were more variable.

**Figure 2:**
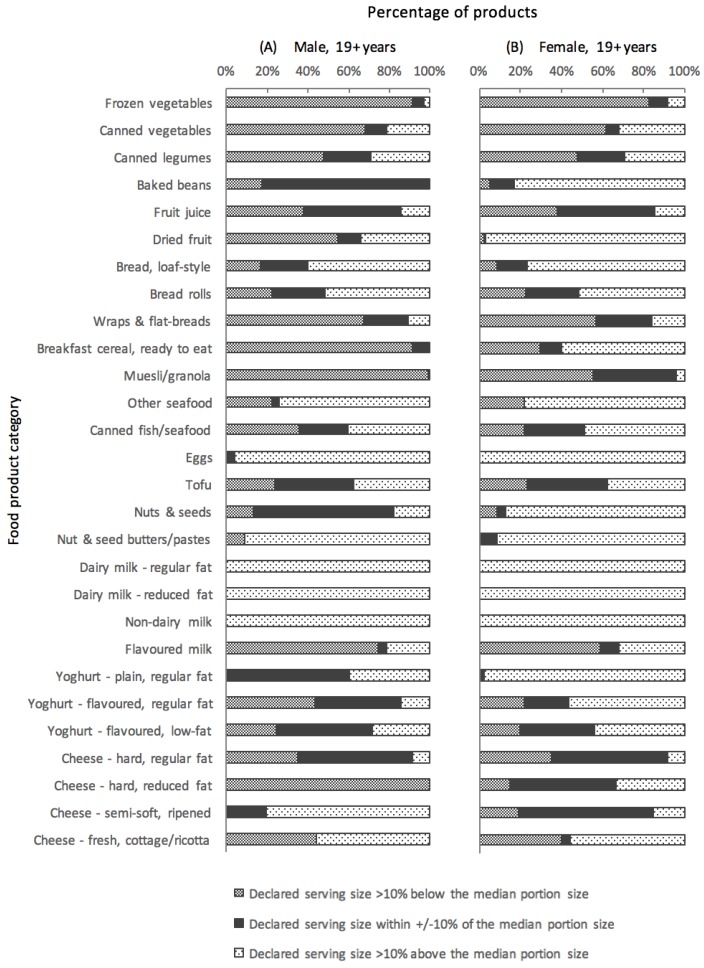
Distribution of products within each food category according to the similarity of their declared serving size to the corresponding typical (median) portion size consumed by Australian adults, for males (**A**) and females (**B**). Note: the category ‘Breakfast cereal, ready to eat’ excludes children’s cereals and muesli/granola.

Figure 2 displays the distribution of products within each category according to the similarity of their declared serving size to sex-specific typical (median) portion sizes consumed by Australian adults. Four out of 28 categories for males and three out of 28 categories for females contained a majority of products with a declared serving size similar to the typical portion size. In contrast, 13 out of 28 categories for males, and 14 out of 28 categories for females, contained very few products with serving sizes similar to the typical portion size. Overall, 23% (744/3247) of all products displayed a declared serving size similar to the corresponding typical portion size for females, and 28% (899/3247) for males. For females, declared serving sizes were generally greater than typical portion sizes, while for males this was more variable.

## 4. Discussion

In this analysis of over 4000 packaged food products from the Five Food Groups, substantial variation in declared serving sizes and energy per serving was observed. Within product categories, coefficients of variation ranged from 0% to 59% for declared serving size, and 9% to 64% for energy per serving. Ten-fold differences were observed between the smallest and largest serving sizes within some product categories. In general, larger variations in energy per serving were observed for categories with highly variable serving sizes. In addition, for many categories, declared serving sizes were substantially different to both the ADG standard serves and to typical portion sizes consumed. Only one quarter of all products analysed displayed a serving size similar (within ±10%) to the corresponding ADG standard serve or typical portion size.

These results are consistent with findings in other food categories (i.e., discretionary foods and snack foods) that observed large variations in declared serving sizes within categories, as well as discrepancies with the ADG standard serves [14,15,17]. Our results highlight the general discrepancies between all three sets of measures—declared serving sizes, ADG standard serves, and typical portion sizes—and suggest that in the absence of serving size regulation, manufacturer-declared serving sizes do not have a consistent basis for determination. Large variations in serving size between similar products confounds the usability of this information for all stakeholders, and particularly as a tool for consumers to monitor and/or regulate their dietary intake. In addition, the observed discrepancies between declared serving sizes and the ADG standard serves do not support consumers in efforts to follow dietary recommendations. Standardising declared serving sizes may be one way to help overcome some of these issues [14,15].

Our data shows that declared serving sizes often differs for similar products between brands, and sometimes within the same brand. Serving sizes were also inconsistent between different package sizes of the same item; this was evident for categories such as baked beans, canned fruit, yoghurts and cheeses. For some food categories, serving sizes were largely based on the weight of a discrete unit/s (e.g., two crispbreads, two slices of bread, or one bread roll) that differed across products and brands. For other categories, the variability in declared serving sizes may be partly derived from the way in which these items are consumed—for example, asparagus spears (20 g serving size) compared to canned tomatoes (100–200 g serving size) within the canned vegetables category. Whether different serving sizes are set by manufacturers with the intent of influencing consumer perceptions [21] or for more practical reasons (e.g., easy division within total package size) is unclear. However, it is clear that these variations are confusing for consumers, and should be addressed with appropriate changes to labelling regulation.

In this study, the smallest variations in serving sizes (CV < 10%) were observed for plain dairy and non-dairy milks. This is likely due to the presence of Australian industry agreed standardised serving sizes for beverages—that is, serving size equals total package size if total package size is ≤600 mL, and serving size is 250 mL if total package size is >600 mL [22]. As plain milks are predominantly sold in package sizes of 1 L or greater, declared serving sizes within these categories are largely consistent. On the other hand, flavoured milks and fruit juices are commonly sold in single-serving (≤600 mL) packages as well as larger packs, and therefore have greater variation in declared serving sizes. Aside from chocolate/sugar confectionery, for which the industry agreed standard is 25 g ± 5 g, standardised serving sizes have not been set for any other product categories at present [22].

In the USA and Canada, declared serving sizes are regulated to reflect typical portion sizes consumed [10,11]. However, as portion sizes vary widely, both between sexes and across individuals [18], using typical portion sizes as the basis for serving size regulation may not be helpful for a large proportion of the population who do not consume the typical portion size. Additionally, due to the high likelihood of inaccuracies in portion size estimation and/or intentional misreporting [23,24,25], self-reported portion size data may not provide a true reflection of portion sizes consumed. It has also been shown that many consumers interpret declared serving size as the recommended amount of food to consume at one time [5,26]. However, national health survey results indicate that typical portion sizes do not reflect dietary recommendations [27]. Australians consume excessive amounts of energy from discretionary foods, and have inadequate intake of foods from the Five Food Groups, such as vegetables, fruit, wholegrain cereals and dairy products [27,28]. This can lead to weight gain as well as inadequate intake of key micronutrients [28,29].

In the USA, recent updates to serving size regulations, intended to make serving sizes more reflective of current portion sizes [10], have resulted in an increase in the reference amounts (upon which serving sizes are regulated) for some foods, and a decrease for others. For instance, the reference amount for ice cream has increased from 1/2 cup to 2/3 cup, and for soda from 240 mL to 360 mL, while for yoghurt has decreased from 225 g to 170 g [10,30,31]. Although the effects of these changes on population consumption patterns are not yet clear [26,32], they highlight a potential problem with serving size regulation if portion sizes continue to diverge from dietary recommendations. Given the current focus on promoting improved food choices and appropriate portion sizes, it appears counterintuitive to base serving size regulations on the same component that public health efforts are attempting to address.

An alternative option could be to regulate serving sizes based on the ADG standard serves. Standardising serving sizes according to the dietary guidelines may assist consumers in understanding how their intake aligns with dietary recommendations, and improve consistency and clarity within nutrition education tools. This may help to challenge distorted consumption norms [4,33], and moderate the portion-size effect, where larger portions or packages have been shown to promote increased consumption [34,35]. It is important to note that the ADG standard serves are not intended as a guide for recommendeding portion sizes [36]. For instance, grain/cereal foods are commonly consumed in portions larger than the ADG standard serve [18]. This is acceptable in the context of an overall intake that aligns with the recommended daily number of serves from each food group [16]. However, reviewing the ADG standard serves to reflect more appropriate and realistic amounts of foods could further improve the usability of nutrition labelling. 

Effective standardisation of declared serving sizes may also provide other benefits, such as improving the accuracy of front-of-pack labelling initiatives based on per serving nutrient information [14]. It would also reduce the potential for manufacturer manipulation of serving sizes [21]. For instance, declaring a smaller serving size results in a lower energy per serving, thus consumers may perceive the food as healthier and consume more [37,38].

While there are clear advantages to standardising serving sizes, there are several challenges to creating such a system. For example, standard serves would need to be developed for all food categories, and an acceptable range of variation in serving size would need to be defined. Nonetheless, improved regulations for declared serving sizes should be seen as an integral aspect of public health strategies to improve population nutrition knowledge and consumption behaviours. Further research should be undertaken to better characterise consumer understanding and use of serving size and per serving nutrient information [9]. Any changes to food labelling policy will need to be accompanied by education campaigns to ensure that consumers understand how to interpret and utilise this information correctly and effectively.

To our knowledge, this study is the first to investigate serving size variations in a wide range of packaged foods from the Five Food Groups in Australia. Data for over 4000 products were collected from the major Australian retailers, providing a good representation of the number and range of products available to consumers within the assessed categories. A limitation was that many foods from the Five Food Groups (predominantly vegetables, fruit, and meats) are unpackaged and/or do not display nutrition information; therefore, these food groups are under-represented in the data. Our data reflect the Australian grocery market; thus, results may not be generalisable to other countries.

## 5. Conclusions

The present study demonstrated substantial variation in manufacturer-declared serving sizes of packaged foods from the Five Food Groups in Australia, as well as discrepancies with the ADG standard serves and typical portion sizes consumed. Standardising declared serving sizes may improve the usability of nutrition information on packaged foods. Standardisation based on the dietary guidelines may assist consumers in following dietary recommendations and improve portion size selections. Any changes to labelling regulation should be accompanied by consumer education campaigns to ensure correct understanding and effective use of the information. Future research is needed to determine how best to proceed with addressing the inconsistencies in declared serving sizes and resulting consumer confusion.

## Figures and Tables

**Table 1:** Descriptive statistics for manufacturer-declared serving sizes and energy per serving for 4046 packaged food products identified from four major Australian supermarkets, in 39 categories, across the Five Food Groups.

Product Categories	*n*	Declared Serving Size			Energy (kJ) Per Serving		
		Median (IQR) ^1^	Range ^1^	CV (%)	Median (IQR) ^1^	Range ^1^	CV (%)
**Vegetables**							
Frozen vegetables	138	100 (75, 100)	40–200	30	176 (117, 247)	47–930	63
Canned vegetables	158	100 (75, 135)	20–200	47	178 (122, 218)	22–642	53
Canned legumes	48	80 (75, 100)	60–125	25	352 (267, 415)	199–696	33
Baked beans	41	210 (206, 220)	100–220	19	829 (737, 898)	370–1214	26
**Fruit**							
Frozen fruit	38	150 (100, 150)	100–220	26	330 (224, 379)	135–527	32
Canned fruit	147	125 (113, 135)	35–170	22	305 (269, 366)	111–882	29
Fruit juice	292	250 (200, 250)	125–500	25	433 (370, 503)	202–1001	30
Dried fruit	126	35 (30, 50)	10–75	29	448 (392, 553)	123–893	29
**Grain (Cereal) Foods**							
Bread, loaf-style	184	74 (61, 83)	31–144	27	765 (637, 866)	275–1392	27
Bread rolls	31	80 (69, 90)	37–170	37	885 (656, 1044)	379–1644	36
Wraps & flat-breads	86	51 (45, 70)	21–105	32	623 (533, 858)	225–1082	32
Breakfast cereal, ready to eat ^2^	171	40 (30, 42)	14–50	22	603 (488, 648)	225–960	22
Breakfast cereal, children’s	44	30 (30, 30)	25–45	13	483 (474, 497)	388–797	13
Muesli/granola	178	45 (45, 50)	30–100	13	799 (731, 869)	380–1560	20
Porridge oats (dry)	72	40 (35, 46)	30–100	25	631 (560, 731)	435–1530	23
Crispbreads	114	21 (12, 25)	3–38	40	371 (206, 463)	60–650	42
**Meat and Alternatives**							
Fish, raw	49	140 (125, 150)	100–187	15	1090 (608, 1236)	200–1590	40
Fish, smoked/cured	41	50 (50, 50)	25–100	30	440 (358, 464)	110–944	34
Other seafood	27	94 (75, 100)	10–150	38	458 (280, 555)	36–1179	58
Canned fish/seafood	320	80 (70, 95)	4–125	24	494 (365, 605)	35–1110	38
Eggs	72	100 (90, 104)	55–118	14	570 (503, 581)	378–672	11
Tofu	90	100 (100, 150)	13–350	51	526 (395, 671)	110–2888	53
Meat substitutes	76	85 (75, 100)	25–150	29	672 (519, 843)	136–1140	35
Nuts & seeds	273	30 (30, 30)	10–100	28	780 (738, 875)	140–2442	31
Nut & seed butters/pastes	78	20 (20, 20)	10–32	21	512 (485, 564)	240–857	22
**Dairy and Alternatives**							
Dairy milk—regular fat	76	250 (250, 250)	150–250	6	674 (656, 702)	389–785	9
Dairy milk—reduced fat	88	250 (250, 250)	200–250	3	466 (380, 489)	304–625	16
Non-dairy milk	44	250 (250, 250)	250–250	0	513 (310, 610)	173–753	38
Flavoured milk	66	250 (250, 425)	150–600	38	868 (689, 1378)	372–2166	43
Yoghurt—plain, regular fat	45	100 (100, 125)	90–200	24	509 (380, 639)	286–1080	33
Yoghurt—plain, low-fat	17	100 (100, 175)	100–200	29	338 (231, 467)	220–590	36
Yoghurt—flavoured, regular fat	152	140 (120, 160)	70–200	22	721 (565, 861)	331–1344	26
Yoghurt—flavoured, low-fat	116	150 (150, 175)	100–200	16	539 (390, 632)	237–740	28
Yoghurt, children’s	64	90 (70, 109)	70–150	28	334 (252, 429)	188–555	31
Cheese—hard, regular fat	217	25 (21, 25)	10–100	42	405 (340, 430)	158–1500	37
Cheese—hard, reduced fat	27	21 (20, 25)	15–25	14	260 (201, 350)	164–360	25
Cheese—semi-soft, ripened	78	25 (25, 25)	20–30	12	379 (326, 408)	240–540	17
Cheese—fresh, cottage/ricotta	25	100 (25, 125)	25–125	59	355 (149, 454)	103–848	64
Cheese—fresh, other	137	25 (25, 28)	10–100	36	328 (259, 371)	82–1164	43
**Total**	4046						

^1^ All units in grams (g) except for fruit juice and milks (regular fat dairy, reduced fat dairy, non-dairy, flavoured), which are in millilitres (mL). ^2^ Excludes children’s cereals and muesli/granola, which were assessed as separate categories. IQR = interquartile range; CV = coefficient of variation. All values rounded to the nearest whole number.

**Table 2:** Comparison of manufacturer-declared serving sizes with the Australian Dietary Guidelines (ADG) standard serves [16] and sex-specific typical (median) portion sizes consumed by Australian adults [18], for 37 categories of packaged food products across the Five Food Groups.

Product Categories	Declared Serving Size		ADG Standard Serves		Typical Portion Sizes			
					Male, 19+ Years		Female, 19+ Years	
	*n*	Median ^1^	Standard Serve ^1^	Percent Difference ^2^	Median ^1^	Percent Difference ^2^	Median ^1^	Percent Difference ^2^
**Vegetables**								
Frozen vegetables	138	100	75	33	143	−30	114	−12
Canned vegetables	158	100	75	33	143	−30	114	−12
Canned legumes	48	80	75	7	87	−8	86	−7
Baked beans	41	210	75	180	201	4	138	52
**Fruit**								
Frozen fruit	38	150	150	0				
Canned fruit	147	125	150	−17				
Fruit juice	292	250	125	100	260	−4	250	0
Dried fruit	126	35	30	17	40	−13	16	119
**Grain (Cereal) Foods**								
Bread, loaf-style	184	74	40	85	64	16	54	37
Bread rolls	31	80	40	100	69	16	69	16
Wraps & flat-breads	86	51	40	28	71	−28	66	−23
Breakfast cereal, ready to eat ^3^	171	40	30	33	51	−22	35	14
Breakfast cereal, children’s	44	30	30	0				
Muesli/granola	178	45	30	50	87	−48	52	−13
Crispbreads	114	21	35	−40				
**Meat and Alternatives**								
Fish, raw	49	140	115	22				
Fish, smoked/cured	41	50	100	−50				
Other seafood	27	94	100	−6	72	31	66	42
Canned fish/seafood	320	80	100	−20	80	0	76	5
Eggs	72	100	120	−17	51	96	49	104
Tofu	90	100	170	−41	100	0	105	−5
Nuts & seeds	273	30	30	0	28	7	27	11
Nut & seed butters/pastes	78	20	30	−33	13	54	10	100
**Dairy and Alternatives**								
Dairy milk—regular fat	76	250	250	0	70	257	50	400
Dairy milk—reduced fat	88	250	250	0	80	213	55	355
Non-dairy milk	44	250	250	0	178	40	127	97
Flavoured milk	66	250	250	0	453	−45	350	−29
Yoghurt—plain, regular fat	45	100	200	−50	92	9	83	20
Yoghurt—plain, low-fat	17	100	200	−50				
Yoghurt—flavoured, regular fat	152	140	200	−30	154	9	123	14
Yoghurt—flavoured, low-fat	116	150	200	−25	156	−4	149	1
Yoghurt, children’s	64	90	200	−55				
Cheese—hard, regular fat	217	25	40	−38	25	0	25	0
Cheese—hard, reduced fat	27	21	40	−48	28	−25	21	0
Cheese—semi-soft, ripened	78	25	40	−38	20	25	24	4
Cheese—fresh, cottage/ricotta	25	100	120	−17	89	12	40	150
Cheese—fresh, other	137	25	40	−38				

^1^ All units in grams (g) except for fruit juice and milks (regular fat dairy, reduced fat dairy, non-dairy, flavoured), which are in millilitres (mL). ^2^ Percent difference calculated as (median serving size − [standard serve OR median portion size])/(standard serve OR median portion size) × 100. ^3^ Excludes children’s cereals and muesli/granola, which were assessed as separate categories. All values rounded to the nearest whole number. Blank cells indicate categories for which no equivalent National Nutrition and Physical Activity Survey (NNPAS) category was identified.

## Appendix A

**Table A1:** Summary of product types included and/or excluded in each product category analysed.

Product Categories	Product Types Included/Excluded
Frozen vegetables	Includes all plain and lightly seasoned frozen vegetables, including minted peas. Excludes frozen potato products, vegetables with grains, vegetable bake (e.g., cauliflower, broccoli), vegetables with cheese sauce.
Canned vegetables	Includes all varieties of canned vegetables—added or no added salt/sugar, canned tomatoes, pickled beetroot, and dried vegetables with nutrition information given for rehydrated product.
Canned legumes	All varieties of canned legumes, both salted and unsalted. Examples include chickpeas, lentils, cannellini beans, kidney beans, black beans, butter beans, four bean mix.
Baked beans	All varieties of canned baked beans—in tomato, cheesy tomato, ham, BBQ, or other flavoured sauce; baked beans and bacon, baked beans and sausages.
Frozen fruit	Includes all frozen whole, diced, pureed frozen fruit, and smoothie mixes, e.g., frozen berries, mango, pineapple, banana, mixed fruit, acai puree, tropical smoothie mix.
Canned fruit	All varieties of canned fruit—in juice or syrup; whole, halved, sliced, diced/pieces, crushed, pulp, puree. Also includes fruit cups in juice or syrup base. Excludes fruit in jelly, fruit and custard.
Fruit juice	Includes all no-added-sugar fruit juices, 100% sparkling fruit juice, juice-based fruit smoothies, fruit and vegetable juice blends with majority (≥50%) fruit juice. Includes both chilled and shelf stable juices—bottled, Tetra Pak, pop-top etc. Excludes vegetable juices, coconut water, fruit and vegetable juice blends with majority (>50%) vegetable juice.
Dried fruit	Includes all regular dried fruit, sweetened dried fruit (e.g., cranberries, mango, pineapple), glacé cherries, banana chips. Excludes freeze-dried fruit/fruit crisps, trail mix, chocolate-/yoghurt-coated dried fruit, mixed peel.
Bread, loaf-style	All white, wholemeal, multi-grain, rye, and gluten free bread loaves—including regular sliced and unsliced bread, sourdough, fruit bread, sandwich thins, pumpernickel, Turkish bread, baguette, French stick. Excludes brioche bread.
Bread rolls	All white, wholemeal, multi-grain, rye, and gluten free bread rolls—including regular dinner rolls, burger buns, hot-dog buns, damper rolls, Turkish bread rolls, bagels.
Wraps & flat-breads	Includes wraps, flat-breads (pita bread, Lebanese bread), tortillas, naan, roti, chappati. Excludes pizza bases, pappadums.
Breakfast cereal, ready to eat (excluding children’s cereals and muesli/granola)	Includes cereal flakes and other extruded cereals, puffed cereals, mixed flakes and clusters, bran sticks, Weet-Bix, and any of the aforementioned with added nuts/seeds/fruit. Excludes plain wheat/oat bran, wheat germ, breakfast biscuits, e.g., Belvita, Weet-Bix Go.
Breakfast cereal, children’s	Breakfast cereals marketed at children; indicated by packaging displaying cartoon/fantasy characters or brand mascots, statements referring to, e.g., “kids” or “children”, referral to childhood themes, e.g., sports, and/or use of language aimed at children.
Muesli/granola	Includes products labelled as “muesli” or “granola”, and cereal clusters without added flakes. Muesli includes natural, toasted, and Bircher varieties.
Porridge oats (dry)	Includes products labelled as “porridge” or “oatmeal”, products displaying porridge images or with preparation directions on packaging, dry oats (rolled oats, quick oats, steel cut oats) and quick oat sachets—both unflavoured and flavoured varieties. Excludes ready-to-eat porridge, porridge oats with nutrition information given only for prepared product.
Crispbreads	Includes all crispbreads, wholemeal or wholegrain wheat/rye/rice crackers (e.g., Vita-Weat and similar products, brown rice crackers), water crackers, wafer crackers, plain lavosh, plain rice/corn cakes, Ryvita cracker bread, SAO crackers, melba toast. Excludes plain refined snack crackers (e.g., Ritz, Jatz), grissini/breadsticks, pastry twists, pita/bagel crisps, cheese or other *flavoured* biscuits, crackers, rice/corn cakes, lavosh.
Fish, raw	Includes all fresh and frozen (uncooked) fish—plain, marinated, or with sauce/dressing. Excludes battered/crumbed fish, and fish products, e.g., fish patties/cakes, fish balls, fish paste, dried salted fish, and roe/caviar.
Fish, smoked/cured	Includes all chilled hot- and cold-smoked, ‘wood roasted’, salt-cured, and pickled fish—primarily salmon, trout and herring.
Other seafood	Includes all fresh, frozen, and ready-to-eat seafood—plain, marinated, with sauce/dressing. Excludes battered/crumbed seafood, and seafood products, e.g., surimi, shrimp paste, seafood salad.
Canned fish/seafood	Includes all canned fish and seafood—in water, brine, oil, sauce; both unflavoured and flavoured, e.g., tuna, salmon, anchovies, mackerel, sardines, mussels, oysters. Excludes ‘snack packs’ of canned fish with crackers, fish ready meals, i.e., with rice/beans.
Eggs	All whole chicken, duck, or quail eggs—raw, cooked/boiled, preserved, salted.
Tofu	All tofu and tempeh—firm, soft, silken, fried, smoked, and flavoured/marinated varieties
Meat substitutes	All chilled, frozen, and canned meat alternative or substitute products. Examples include vegetarian patties and burger patties, mince, nuggets, meat-free strips/pieces, fillets, schnitzel, ‘fish’ fingers, sausages, hot dogs, veggie ‘roast’, deli slices, bacon, falafels and other vegetable bites.
Nuts & seeds	Includes all raw, blanched, dry-roasted, oil-roasted, unsalted, salted, smoked, seasoned/flavoured nuts and seeds—whole, halves, pieces, flaked, slivered, ground (e.g., almond meal). Includes LSA, mixed nuts and/or seeds. Excludes coconut products, trail mix, snack mixes containing nuts/seeds, coated nuts (e.g., sugar-coated, chocolate-coated, deli-style crispy coated nuts).
Nut & seed butters/pastes	All spreads/pastes consisting of a majority of ground nuts/seeds, e.g., peanut, almond, cashew, brazil nut, sesame seed (tahini). Includes all varieties—smooth, crunchy, unsalted, salted, unsweetened, sweetened, with added oil, flavoured (e.g., chocolate, honey, cinnamon), added grains. Excludes nut/seed spreads consisting of <50% nuts/seeds.
Dairy milk—regular fat	Includes all full cream fresh, UHT, and powdered dairy milks*. ‘Dairy’ includes cow, goat, and sheep milks. Includes lactose-free varieties. * Excludes powdered milk products for which serving size and nutrition information are given only for dry powder. Note: all products included contained >3% fat.
Dairy milk—reduced fat	Includes all reduced-fat, semi-skim, skim, ‘lite’/light, and no-fat fresh, UHT, and powdered dairy milks* and buttermilk. ‘Dairy’ includes cow, goat, and sheep milks. Includes lactose-free varieties. * Excludes powdered milk products for which serving size and nutrition information are given only for dry powder. Note: all included products contained ≤2% fat.
Non-dairy milk	All plain (unflavoured) alternative/non-dairy milks with at least 100 mg/100 mL of added calcium [16]. Varieties include soy, rice, oat, almond, coconut. Includes all regular fat, reduced fat, unsweetened and sweetened varieties.
Flavoured milk	Includes dairy and non-dairy flavoured milks, milkshakes, milk-based iced coffee and smoothies, and other milk-based drinks with at least 100 mg/100 mL of calcium (labelled). Includes lactose-free varieties.
Yoghurt—plain, regular fat	Includes all plain/natural, unsweetened yoghurts with ≥2.5% fat. Includes soy yoghurt with added calcium.
Yoghurt—plain, low-fat	As above, but varieties with <2.5% fat.
Yoghurt—flavoured, regular fat	Includes all flavoured and fruit yoghurts with ≥*2.5% fat.* Includes soy yoghurt with added calcium. Excludes yoghurt with added grains, oats, muesli, nuts, seeds, biscuit pieces, etc., and non-dairy yoghurts without added calcium.
Yoghurt—flavoured, low-fat	As above, but varieties with *<2.5% fat.*
Yoghurt, children’s	Yoghurt marketed at children; indicated by packaging displaying cartoon/fantasy characters or brand mascots, statements referring to, e.g., “kids”, “children”, “lunch boxes”, referral to childhood themes, e.g., sports, and/or use of language aimed at children.
Cheese—hard, regular fat	Varieties include Parmesan, Grana Padano, Cheddar (mild, tasty, sharp), Edam, Babybel, Colby, Swiss, Emmental, Jarlsberg, Maasdam, manchego, provolone, Gruyere, Gouda, Red Leicester, processed cheese (slices, sticks, string cheese). Includes both plain and flavoured varieties. Includes soy-based cheeses with added calcium, and pizza blends with >50% cheddar cheese. Excludes non-dairy cheese without added calcium.
Cheese—hard, reduced fat	As above, but labelled as ‘light’/‘lite’, ‘reduced fat’, or otherwise indicating reduced fat content.
Cheese—semi-soft, ripened	Varieties include brie, camembert, Havarti, blue cheese (including Gorgonzola, Stilton)—both plain and flavoured varieties.
Cheese—fresh, cottage/ricotta	Includes cottage cheese, ricotta, and quark—both regular fat and reduced fat, plain and flavoured varieties.
Cheese—fresh, other	Other fresh unripened cheeses. Varieties include cream cheese, mascarpone, spreadable cheese, feta (fetta), soft goat and sheep cheeses, mozzarella, bocconcini, burrata, halloumi—both regular fat and reduced fat, plain and flavoured. Includes fruit and nut cream cheeses, soy-based cheeses with added calcium, pizza blends consisting of >50% mozzarella cheese. Excludes crumbed cheese, and non-dairy cheese without added calcium.

**Table A2:** Product categories analysed and names of corresponding categories [18] used for comparisons to typical portion sizes consumed by Australian adults.

Product Categories	Corresponding Categories
Frozen vegetables	Mixed vegetables, nonleafy
Canned vegetables	Mixed vegetables, nonleafy
Canned legumes	Cooked legumes and pulses
Baked beans	Baked beans, canned
Fruit juice	Fruit juices
Dried fruit	Sultanas/Raisins
Bread, loaf-style	Bread, white
Bread rolls	Rolls, white
Wraps & flat-breads	Flat-breads
Breakfast cereal, ready to eat	Breakfast cereal, ready to eat
Muesli/granola	Breakfast cereal, muesli, untoasted
Other seafood	Other seafood, cooked
Canned fish/seafood	Fish and seafood, canned
Eggs	Eggs, whole
Tofu	Meat alternatives (tofu)
Nuts & seeds	Nuts, whole
Nut & seed butters/pastes	Peanut butter
Dairy milk—regular fat	Milk—full fat
Dairy milk—reduced fat	Milk—reduced fat
Non-dairy milk	Milk substitutes
Flavoured milk	Flavoured milk
Yoghurt—plain, regular fat	Yoghurt—plain
Yoghurt—flavoured, regular fat	Yoghurt—flavoured, full fat
Yoghurt—flavoured, low-fat	Yoghurt—flavoured, reduced fat
Cheese—hard, regular fat	Cheese, cheddar-type, full fat
Cheese—hard, reduced fat	Cheese, cheddar-type, reduced fat
Cheese—semi-soft, ripened	Cheese, brie or camembert
Cheese—fresh, cottage/ricotta	Cheese, cottage or ricotta
